# Spectroscopic data of 6-(*N*-methyl-pyridin-2-ylcarbamoyl)-pyridine-2-carboxylic acid methyl ester isomers

**DOI:** 10.1016/j.dib.2019.104266

**Published:** 2019-07-16

**Authors:** M.A. Kadir, N. Mansor, M.U. Osman, N.S.H. Haris

**Affiliations:** School of Fundamental Science, Universiti Malaysia Terengganu, 21030 Kuala Nerus, Malaysia

**Keywords:** Monoamide, Ligand, Acyl chloride, Isomers

## Abstract

This paper provided spectroscopic data that is relevant with research article entitled “Synthesis and structural characterization of 6-(*N*-methyl-pyridin-2-ylcarbamoyl)-pyridine-2-carboxylic acid methyl ester Isomer” (Kadir et al., 2017) [1]. From the reported study, four new ligand of monoamide isomers were successfully synthesized using acyl chloride methods. The monoamide compounds namely 6-(3-methyl-pyridin-2-ylcarbamoyl)-pyridine-2-carboxylic acid methyl ester (L1), 6-(4-methyl-pyridin-2-ylcarbamoyl)-pyridine-2-carboxylic acid methyl ester (L2), 6-(5-methyl-pyridin-2-ylcarbamoyl)-pyridine-2-carboxylic acid methyl ester (L3) and 6-(6-methyl-pyridin-2-ylcarbamoyl)-pyridine-2-carboxylic acid methyl ester (L4) were fully characterized by Fourier Transform Infrared (FTIR), ^1^H Nuclear Magnetic Resonance (^1^H NMR) and ^13^C Nuclear Magnetic Resonance (^13^C NMR), Ultraviolet Visible (UV–Vis) and elemental analyzer (CHNS).

**Table:** Specification table

Subject area	*Chemistry*
More specific subject area	*Synthetic chemistry, spectroscopy*
Type of data	*FTIR spectra, NMR spectra, UV spectra, graph, table*
How data was acquired	CHNS Analyzer Flashea 1112 series, FTIR Perkin Elmer Spectrum 100 and the spectra was recorded in range of 4000–400 cm^−1^ utilizing potassium bromide (KBr) pellet, Spectrophotometer Shimadzu UV-1800, Bruker Avance II 400 spectrometer was used to record the ^1^H and ^13^C Nuclear Magnetic Resonance
Data format	*JPEG, Tiff (Raw)*
Experimental factors	Product was isolated using column chromatography and obtained as pale yellow precipitate. For NMR and UV Vis analysis, sample was dissolved in suitable solvent.
Experimental features	*All chemicals used were commercially available and used as received without purification.*
Data source location	*Universiti Malaysia Terengganu*
Data accessibility	*Data is included with this article*
Related research article	M.A. Kadir*,N. Mansor, M.U. Osman, Synthesis and Structural Characterization of 6-(N-methyl-pyridin-2-ylcarbamoyl)-pyridine-2-carboxylic acid methyl ester Isomer, Sains Malaysiana, (2017), 46(5), 725 – 731.

**Table:** 

**Value of the data** • The data obtained from combination of FTIR, NMR and UV–Vis spectroscopic methods is useful in structure characterization and confirmation of new molecules. • Chemical database that specifically related with methyl ester derivatives is developed from this research. • The details in the experimental data are important to produce amino pyridine derivatives for potential used in hydrogen storage.

## 1 Data

Four new compounds namely 6-(3-methyl-pyridin-2-ylcarbamoyl)-pyridine-2-carboxylic acid methyl ester (L1), 6-(4-methyl-pyridin-2-ylcarbamoyl)-pyridine-2-carboxylic acid methyl ester (L2), 6-(5-methyl-pyridin-2-ylcarbamoyl)-pyridine-2-carboxylic acid methyl ester (L3) and 6-(6-methyl-pyridin-2-ylcarbamoyl)-pyridine-2-carboxylic acid methyl ester (L4) were synthesized from reaction between 6-(methoxycarbonyl)pyridine-2-carboxylic acid and aminomethylpyridine in dichloromethane [1]. These compounds were varied by different placements of methyl substituents at ortho, meta and para. Acyl chloride method was selected to enhance the nucleophilicity of aminopyridin in the reaction [2], [3].

## 2 Experimental design, materials, and methods

A suspension of 6-(methoxycarbonyl)pyridine-2-carboxylic acid (0.5 g, 2.0 mol), thionyl chloride (0.5 mL) and dried DMF (1 μL) was refluxed in dichloromethane (100 mL). After an hour, the dichloromethane was removed using rotary evaporator to remove the solvent. The obtaining white solid (1.67 g, 3.5 mol) was redissolved in dichloromethane (40 mL) before added with 2-amino-3-methyl pyridine (1.567 g, 3.5 mol). The mixture was continued to reflux for another 24 h. After the reaction was completed, the solvent was removed using rotary evaporator. Then, the residue was dissolved in dichloromethane and washed with sodium hydrogen bicarbonate. The residue was dried over magnesium sulfate before being removed by rotavap. The residue was further purified by column chromatography on silica gel eluting with 8:2 ethyl acetate: dichloromethane to give product as pale yellow precipitate of 6-(3-methyl-pyridin-2-ylcarbamoyl)-pyridine-2-carboxylic acid methyl ester (L1). Compound L1 was obtained as yellow precipitate*.* The rest of the compounds (L2-L4) were prepared using similar methods described for L1, by replacing 2-amino-3-methyl pyridine with 2-amino-4-methyl pyridine, 2-amino-5-methyl pyridine and 2-amino-6-methyl pyridine, respectively (see Table 1, Table 2, Table 3, Table 4, Table 5).

**Table 1:** The FTIR spectra data for all four monoamide ligands, L1, L2, L3 and L4.

Vibrational modes	L1 (cm^−1^)	L2 (cm^−1^)	L3 (cm^−1^)	L4 (cm^−1^)
ν(CH_3_)	2925	2923	2962	2920
ν(N–H str)	3339	3357	3350	3358
ν(C=O)	1732, 1702	1742, 1727	1731, 1702	1725, 1699
ν(N–H bend)	1567, 1535	1533	1533	1525
ν(CH_3_ bend)	1324	1321	1322	1320
ν(O–CH_3_) str	1144	1133	1133	1133
ν(C–N)	1071	1075	1076	1076
ν(C=N)	1613	1583	1583	1583

**Table 2:** ^1^H NMR (a) L1, (b) L2, (c) L3, (d) L4.

Compound	^1^H NMR (δ_,_ ppm)	
L1	2.39	3H, s, δ (Py-CH_3_)
	4.02	3H, s, δ (O–CH_3_)
	7.15	1H, d, δ (py-H)
	7.61	1H, t, 7 Hz, δ (py-H)
	8.06	1H, d, 7.7 Hz, δ (py-H)
	8.29	1H, d, 7.7 Hz, δ (py-H)
	8.37	1H, d, 4.9 Hz, δ ((py-H)
	8.48	1H, d, 7.7 Hz, δ (py-H)
	10.28	1H, s, δ ((N–H)
L2	2.45	3H, s, δ (Py-CH_3_)
	4.06	3H, s, δ (O–CH_3_)
	6.97	1H, d, 4.9 Hz, δ (py-H)
	8.09	1H, t, 7.7 Hz, δ (py-H)
	8.26	1H, d, 4.9 Hz, δ (py-H)
	8.31	2H, d, 8.4 Hz, δ (py-H)
	8.49	1H, d, 7.7 Hz, δ ((py-H)
	10.28	1H, s, δ ((N–H)
L3	2.36	3H, s, δ (Py-CH_3_)
	4.06	3H, s, δ (O–CH_3_)
	7.61	1H, d, 7.7 Hz, δ (py-H)
	8.08	1H, t, 7.7 Hz, δ (py-H)
	8.23	1H, s, δ (py-H)
	8.31	1H, d, 7.7 Hz, δ (py-H)
	8.34	1H, d, 8.4 Hz, δ ((py-H)
	8.49	1H, d, 7.7 Hz, δ (py-H)
	10.41	1H, s, δ ((N–H)
L4	2.53	3H, s, δ (Py-CH_3_)
	4.06	3H, s, δ (O–CH_3_)
	6.96	1H, d, 7.7 Hz, δ (py-H)
	7.66	1H, t, 7.7 Hz, δ (py-H)
	8.07	1H, d, 7.7 Hz, δ (py-H)
	8.21	1H, d, 8.4 Hz, δ (py-H)
	8.28	1H, d, 7.7 Hz, δ ((py-H)
	8.48	1H, d, 7.7 Hz, δ (py-H)
	10.36	1H, s, δ ((N–H)

**Table 3:** ^13^C NMR of (a) L1, (b) L2, (c) L3, (d) L4.

Compound	^13^C NMR (δ_,_ ppm)	
L1	18.07	(py-CH_3_)
	52.95	(py-OCH_3_)
	121.67	(py-C)
	125.75	(py-C)
	127.68	(py-C)
	127.70	(py-C)
	138.81	(py-C)
	139.74	(py-C)
	146.32	(py-C)
	146.60	(py-C)
	149.13	(py-C)
	149.79	(py-C)
	161.27	(C=O)
	164.86	(C=O)
L2	21.55	(py-CH_3_)
	53.03	(py-OCH_3_)
	114.97	(py-C)
	121.38	(py-C)
	125.64	(py-C)
	127.85	(py-C)
	138.82	(py-C)
	146.97	(py-C)
	147.07	(py-C)
	149.49	(py-C)
	150.62	(py-C)
	150.68	(py-C)
	161.91	(C=O)
	164.95	(C=O)
L3	17.93	(py-CH_3_)
	53.00	(py-OCH_3_)
	113.77	(py-C)
	125.59	(py-C)
	127.73	(py-C)
	129.59	(py-C)
	138.76	(py-C)
	138.93	(py-C)
	146.92	(py-C)
	148.13	(py-C)
	148.79	(py-C)
	149.70	(py-C)
	161.70	(C=O)
	164.99	(C=O)
L4	24.13	(py-CH_3_)
	53.01	(py-OCH_3_)
	111.13	(py-C)
	119.70	(py-C)
	125.64	(py-C)
	127.74	(py-C)
	138.59	(py-C)
	138.76	(py-C)
	146.91	(py-C)
	149.76	(py-C)
	150.25	(py-C)
	157.28	(py-C)
	161.83	(C=O)
	164.96	(C=O)

**Table 4:** The result of UV–Vis spectroscopy for L1-L4.

Compound	Chromophores	Transition	*λ*_max_ (nm)	ε, L mol^−1^ cm^−1^
L1	Pyridine, C=O	n → π*, π → π*	273	2.73 × 10^7^
L2	Pyridine, C=O	n → π*, π → π*	273	2.73 × 10^7^
L3	Pyridine, C=O	n → π*, π → π*	293	2.93 × 10^7^
L4	Pyridine, C=O	n → π*, π → π*	291	2.91 × 10^7^

**Table 5:** The elemental analysis data of L1-L4.

Percentage of element			
Compound	%C	%H	%N
L1	62.6	5.1	15.0
L2	58.0	5.0	14.6
L3	61.4	4.7	15.7
L4	61.7	4.8	15.2

**Fig. 1:**
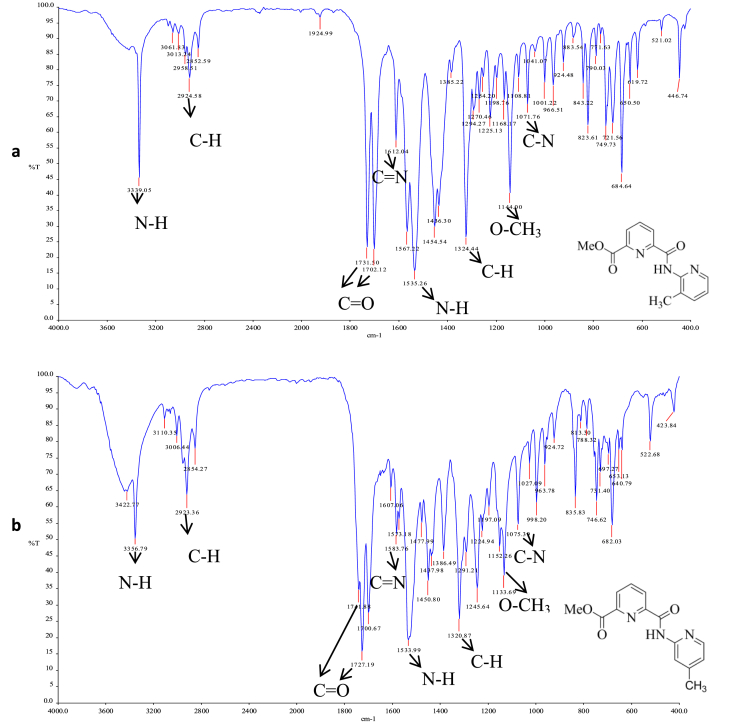

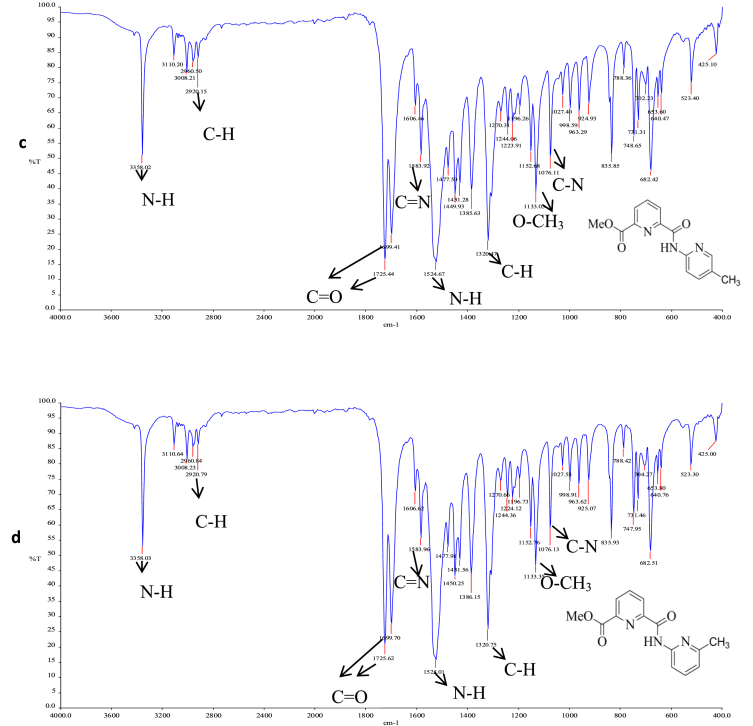
The FTIR spectrum for (a) L1, (b) L2, (c) L3 and (d) L4.

**Fig. 2:**
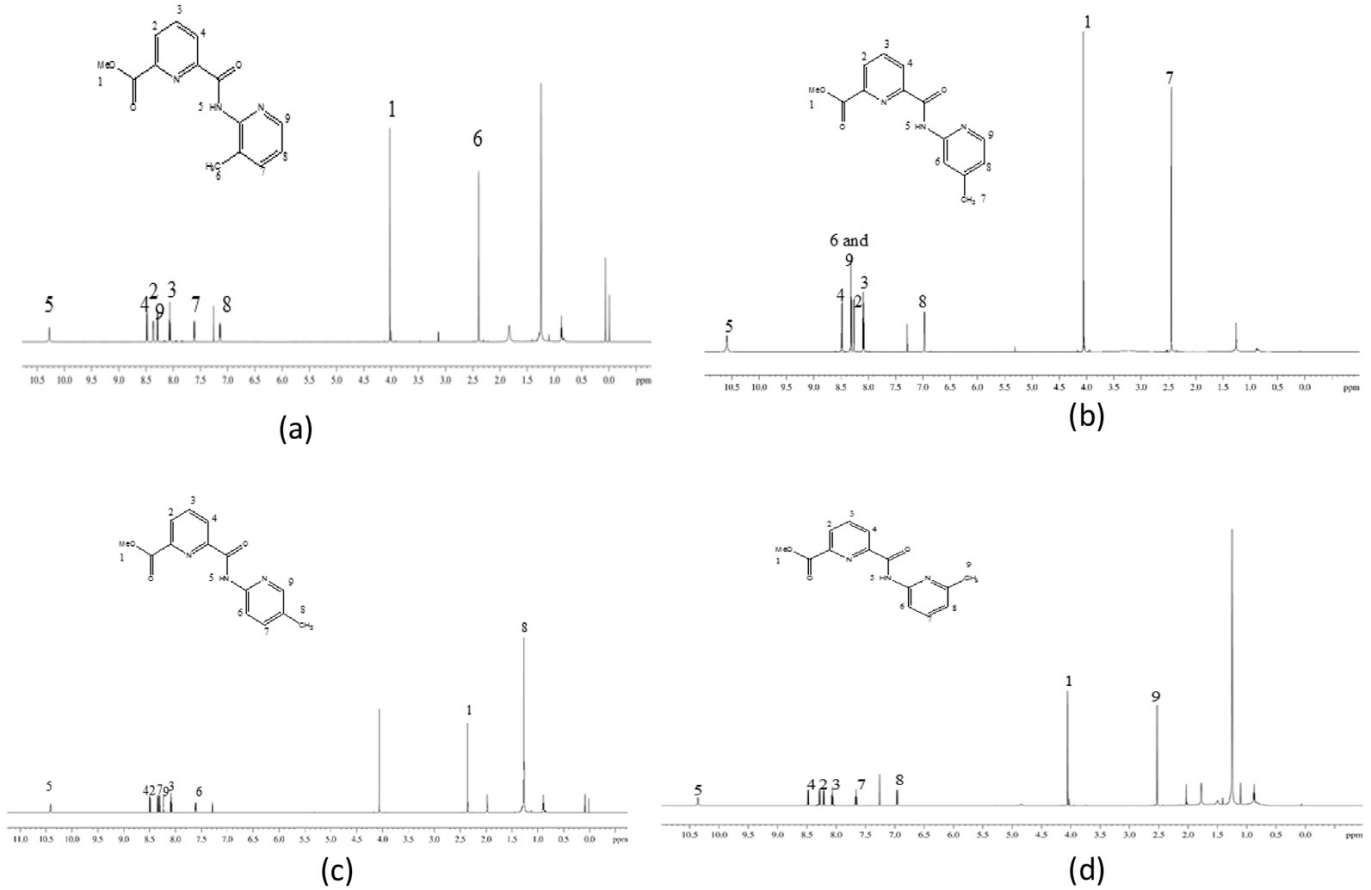
^1^H NMR (a) L1, (b) L2, (c) L3, (d) L4 in DMSO-*d6*.

**Fig. 3:**
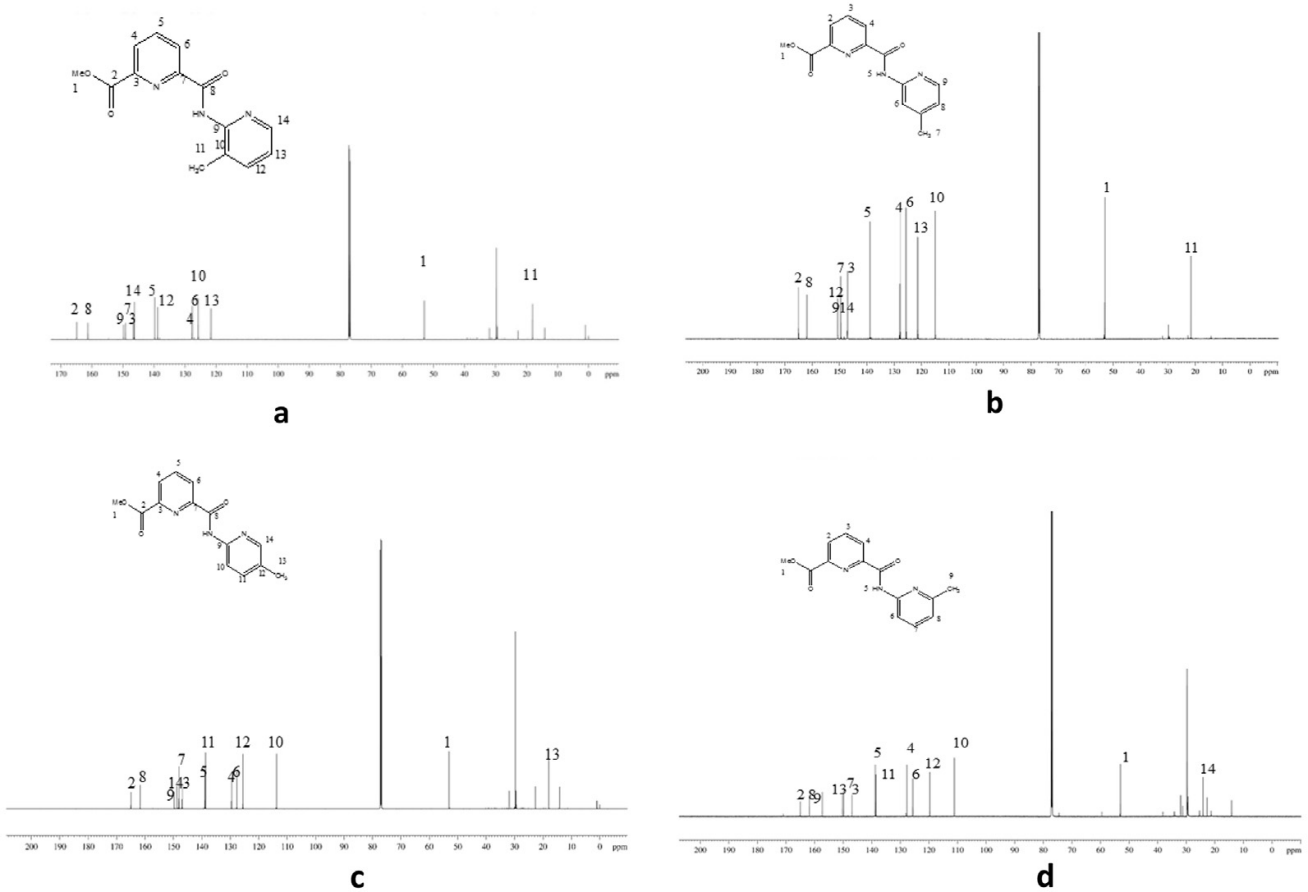
^13^C NMR of (a) L1, (b) L2, (c) L3, (d) L4 in DMSO-*d6*.

**Fig. 4:**
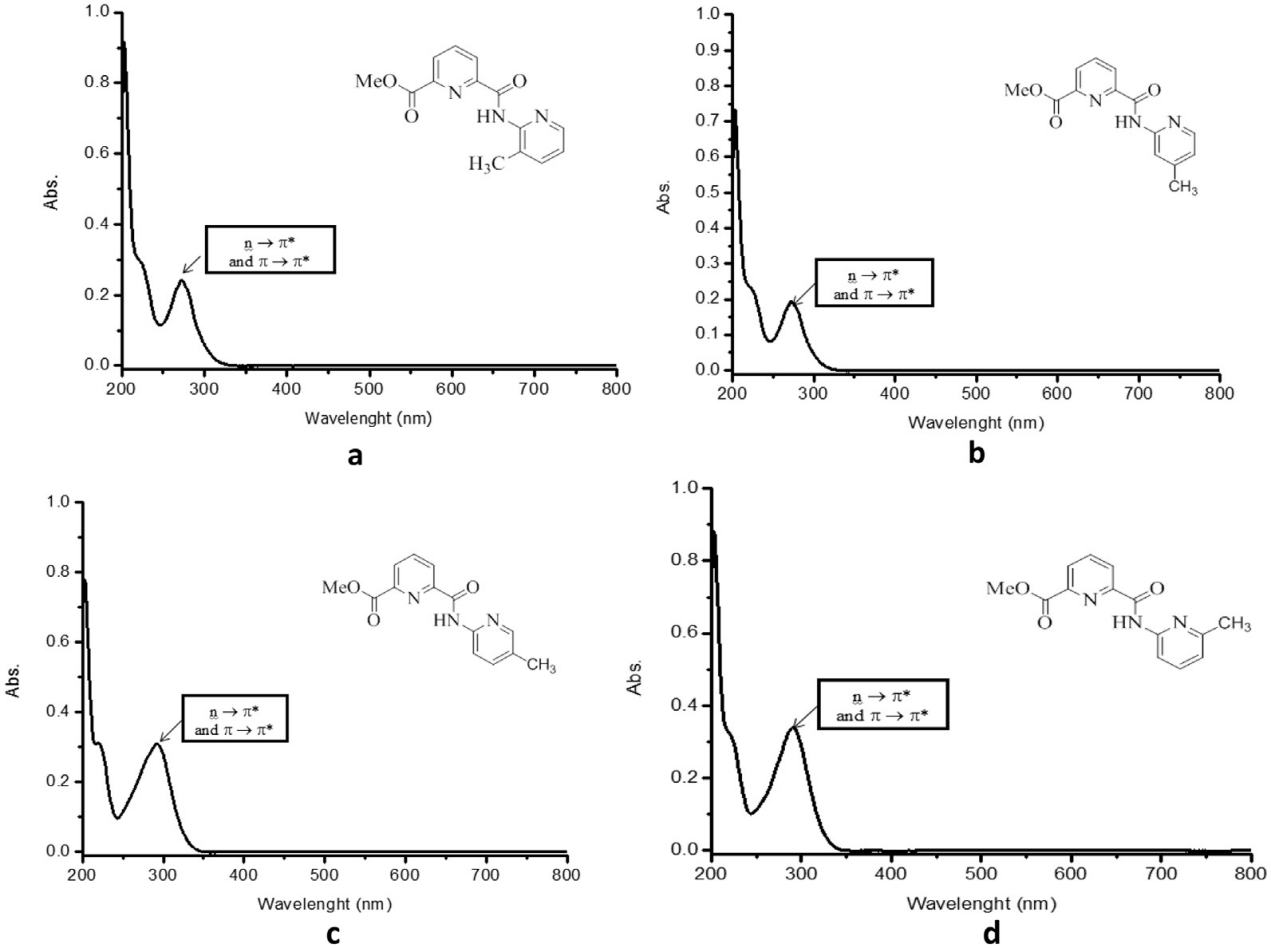
UV spectra of (a)L1, (b)L2, (c)L3, (d)L4 in methanol solution.

The methyl ester derivatives were characterized by using combination of spectroscopic techniques such as FTIR, ^1^H NMR and ^13^C NMR, UV–Vis. The spectroscopic data was supported [4], [5] and are depicted in Fig. 1, Fig. 2, Fig. 3 and Fig. 4, respectively.
